# Supplementary material for: Brown adipose tissue monocytes support tissue expansion
Source: Nat Commun. 2021 Sep 6;12:5255. doi: 10.1038/s41467-021-25616-1 (PMC8421389; doi:10.1038/s41467-021-25616-1)
Supplement: Supplementary file 1 — Supplementary Information [file 41467_2021_25616_MOESM1_ESM.pdf]

Supplementary information for :

Brown adipose tissue monocytes support tissue expansion.

Authors

Alexandre Gallerand[1£], Marion I. Stunault[1£], Johanna Merlin[1], Hannah P. Luehmann[2], Deborah H. Sultan[2], Maria M. Firulyova[3], Virginie Magnone[4], Narges Khedher[1], Antoine Jalil[5], Bastien Dolfi[1], Alexia Castiglione[1], Adelie Dumont[1], Marion Ayrault[1], Nathalie Vaillant[1], Jérôme Gilleron[1], Pascal Barbry[4], David Dombrowicz[6], Matthias Mack[7], David Masson[5], Thomas Bertero[4], Burkhard Becher[8], Jesse W. Williams[9], Konstantin Zaitsev[3], Yongjian Liu[2], Rodolphe R. Guinamard[1], Laurent Yvan-Charvet[1] and Stoyan Ivanov[1*]

Affiliations

[1] Université Côte d'Azur, INSERM, C3M, Nice, France.
[2] Department of Radiology, Washington University School of Medicine, Saint Louis, Missouri 63110 United States.
[3] Computer Technologies Department, ITMO University, Saint Petersburg, Russia.
[4] Université Côte d'Azur, CNRS, IPMC, Valbonne, France.
[5] Université Bourgogne Franche-Comté, LNC UMR1231, F-21000 Dijon, France.
[6] Univ.Lille, Inserm, CHU Lille, Institut Pasteur de Lille, U1011-EGID, 59000 Lille, France.
[7] Department of Internal Medicine - Nephrology, University Hospital Regensburg, Regensburg, Germany.
[8] Institute of Experimental Immunology, University of Zürich, Switzerland.
[9] Department of Integrative Biology and Physiology, Center for Immunology, University of Minnesota Medical School, Minneapolis, MN, USA.

[£]Equal contribution
[*]Corresponding author

Correspondence to:
Stoyan.ivanov@unice.fr

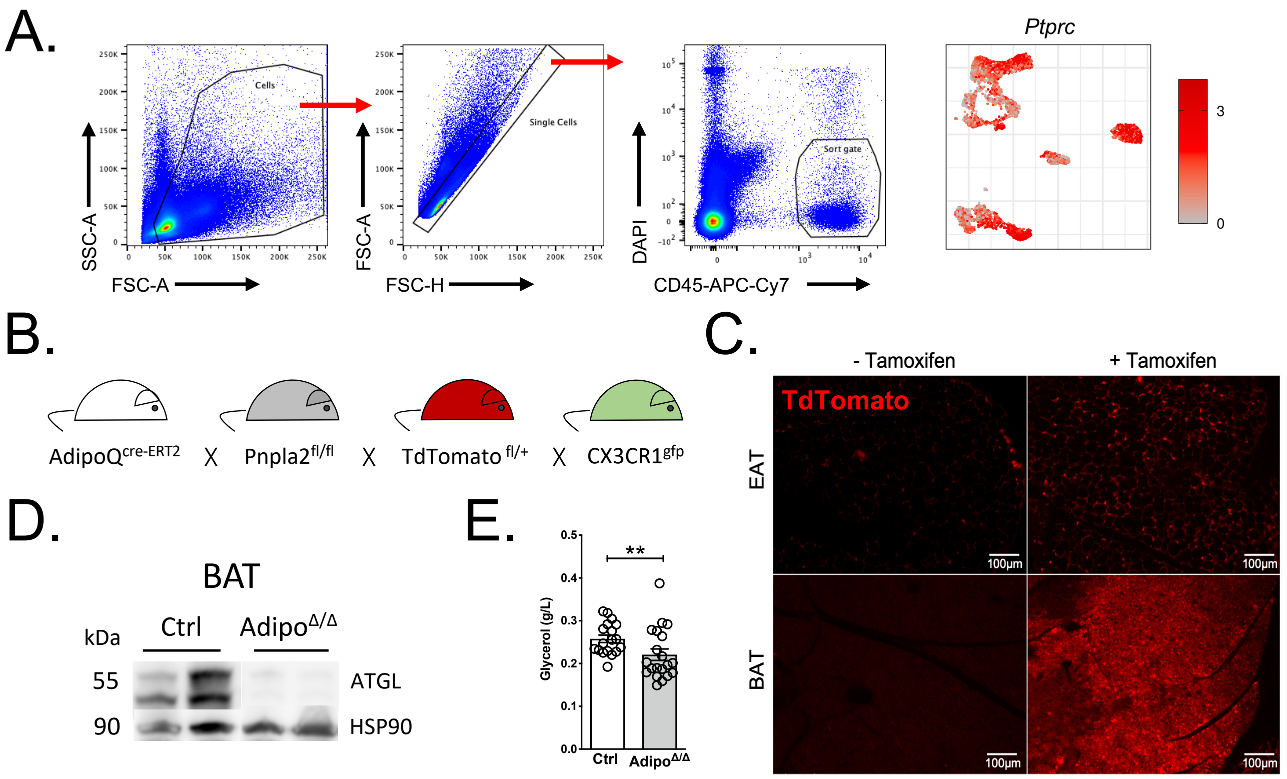

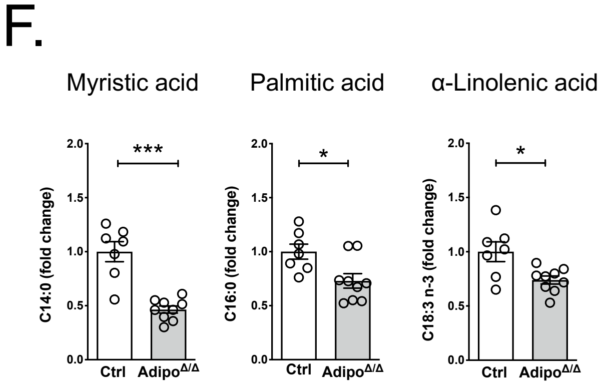

G.

Name	Formula	Ratio compared to ctrl	+/- SEM
Laureic acid	C12:0	1,0437643	0,0457
Myristic acid *	C14:0	0,47300615	0,0328
Palmitoleic acid	C16:1 n-9	0,96329024	0,1434
Methyl palmitoleic acid *	C16:1 n-7	0,57340346	0,07513
Palmitic acid *	C16:0	0,73588931	0,06703
Oleic acid	C18:1 n-9	0,74614637	0,08332
Trans-vaccenic acid	C18:1 n-7	0,93253779	0,1181
Stearic acid	C18:0	0,83793297	0,05683
linoleic acid	C18:2 n-6	0,83610225	0,04359
α-linolenic acid *	C18:3 n-3	0,7386928	0,03713
cis-11-Eicosenoic acid	C20:1 n-9	0,8906383	0,09488
dihomo-γ-linolenic acid	C20:3 n-6	0,92904146	0,1156
Mead acid	C20:3 n-9	0,89803058	0,1329
Arachidonic acid	C20:4 n-6	0,9339235	0,109
Eicosapentaenoic acid	C20:5 n-3	1,00486347	0,1208
Docosahexaenoic acid	C22:6 n-3	0,90164926	0,1014

Figure S1. This figure is related to Figure 1.

(**A**) (Left) Cell-sorting strategy used for isolation of BAT $CD45^+$ cells before scRNA-Seq and (right) Single Cell RNA-Seq analysis of *Ptprc* expression among BAT $CD45^+$ cells (**B**) Breeding scheme for generating Adipo$^{\Delta/\Delta}$ mice. (**C**) TdTomato reporter expression (red) in epididymal (EAT) and brown (BAT) adipose tissues by fluorescence microscopy. (**D**) ATGL protein expression in Adipo$^{\Delta/\Delta}$ mice brown adipose tissue (BAT) compared to controls (Ctrl) by Western Blot analysis; HSP90 was used as loading control for protein expression. (**E**) Glycerol levels in control (n=18) and Adipo$^{\Delta/\Delta}$ (n=20) mice sera. p= 0,0096. (**F**) Myristic acid (C14:0), palmitic acid (C16:0) and α-linoleic acid (C18:3 n-3) levels in control (n=7) and Adipo$^{\Delta/\Delta}$ (n=9) mice sera. p=0,0003 (left), p=0,0115 (middle), p= 0,0418 (right). (**G**) Lipidomic analysis of serum from control and Adipo$^{\Delta/\Delta}$ mice. Data are represented in ratio compared to Ctrl condition ± SEM. Stars indicate a statistically significant difference between controls and Adipo$^{\Delta/\Delta}$ mice (p<0,05).

Panel C is representative of 3 experiments. Panel D represents one experiment. Panel E represents pooled data from 5 independent experiments. Panels F and G represent pooled data from 2 independent experiments. All data are represented in means ± SEM. Two-tailed Mann Whitney tests were used to determine statistical significance in panels E and F. ns p>0,05 ; * p<0,05 ; **<0,01 ; *** p<0,001 ; **** p<0,0001. Source data, and notably uncropped blots for panel S1D, are provided as a Source Data file.

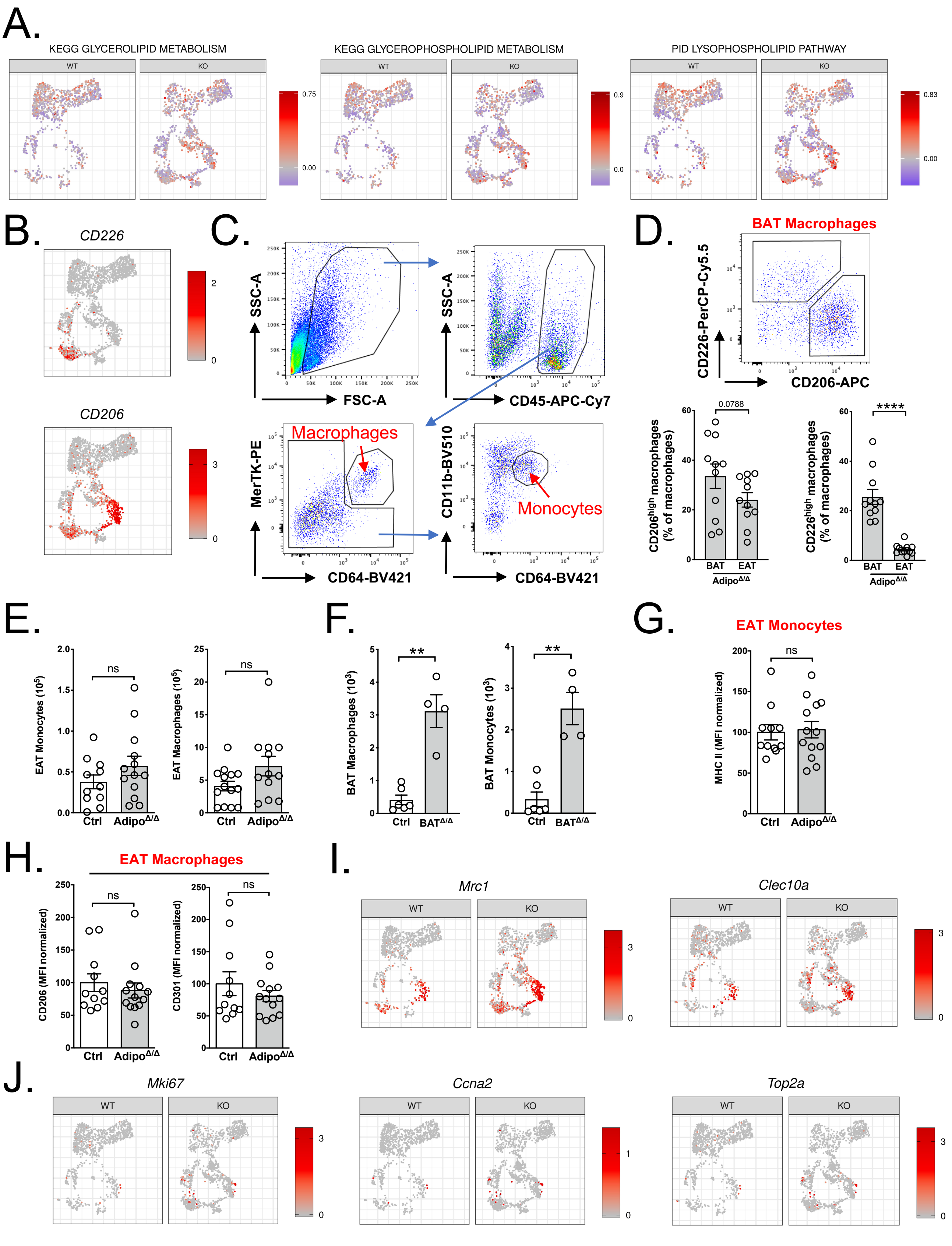

A.
KEGG GLYCEROLIPID METABOLISM
WT
KO
0.75
0.00
KEGG GLYCEROPHOSPHOLIPID METABOLISM
0.9
0.0
PID LYSOPHOSPHOLIPID PATHWAY
0.83
B.
CD226
CD206
C.
SSC-A
FSC-A
CD45-APC-Cy7
MerTK-PE
Macrophages
CD64-BV421
CD11b-BV510
Monocytes
D.
BAT Macrophages
CD226-PerCP-Cy5.5
CD206-APC
CD206high macrophages (% of macrophages)
0.0788
BAT
EAT
AdipoΔ/Δ
CD226high macrophages (% of macrophages)
****
E.
EAT Monocytes (10^5)
EAT Macrophages (10^5)
ns
Ctrl
AdipoΔ/Δ
F.
BAT Macrophages (10^3)
BAT Monocytes (10^3)
**
BATΔ/Δ
G.
EAT Monocytes
MHC II (MFI normalized)
H.
EAT Macrophages
CD206 (MFI normalized)
CD301 (MFI normalized)
I.
Mrc1
Clec10a
J.
Mki67
Ccna2
Top2a

Figure S2. This figure is related to Figure 2.
(**A**) Single Cell RNA-Seq analysis of the PID “lysophospholipid metabolism” pathway, the KEGG “glycerolipid metabolism” gene set and the KEGG “glycerophospholipid metabolism” gene set expression. (**B**) Single Cell RNA-Seq analysis of *Mrc1* and *CD226* expression among BAT monocytes and macrophages. (**C**) Gating strategy used for identification of tissue macrophages, and tissue monocytes in mice lacking the $CX3CR1^{gfp}$ reporter. (**D**) Representative dot plots (left) and proportions (right) of BAT and EAT $CD206^{high}$ and $CD226^{high}$ macrophages in $Adipo^{\Delta/\Delta}$ mice (n=11). *p*=0,0788 (left), *p*<0,0001 (right). (**E**) Quantification of EAT macrophage and monocyte counts in control (n=11) and $Adipo^{\Delta/\Delta}$ (n=16) mice using flow cytometry. *p*=0,3607 (left), *p*=0,1207 (right). (**F**) Quantification of BAT monocyte and macrophage numbers in control (n=6) and $BAT^{\Delta/\Delta}$ (n=4) mice using flow cytometry. *p*= 0,0095 (left and right). (**G**) Quantification of surface MHCII expression on EAT monocytes in control (n=14) and $Adipo^{\Delta/\Delta}$ mice (n=15) using flow cytometry. *p*=0,9095. (**H**) Quantification of surface CD206 and CD301 expression by EAT macrophages in control (n=11) and $Adipo^{\Delta/\Delta}$ (n=16) mice using flow cytometry. *p*=0,6905 (left and right). (**I**) Single Cell RNA-Seq analysis of *Mrc1* (CD206) and *Clec10a* (CD301) expression among BAT myeloid cells. (**J**) Single Cell RNA-Seq analysis of genes involved in cell proliferation.
Panel D represents pooled data from 2 independent experiments. Panels E, G and H represent pooled data from 4 independent experiments. Panel F is representative from 2 independent experieements. All data are represented in means ± SEM. Two-tailed Mann Whitney tests were used to determine statistical significance. ns p>0,05 ; * p<0,05 ; **<0,01 ; *** p<0,001 ; **** p<0,0001. Source data are provided as a Source Data file.

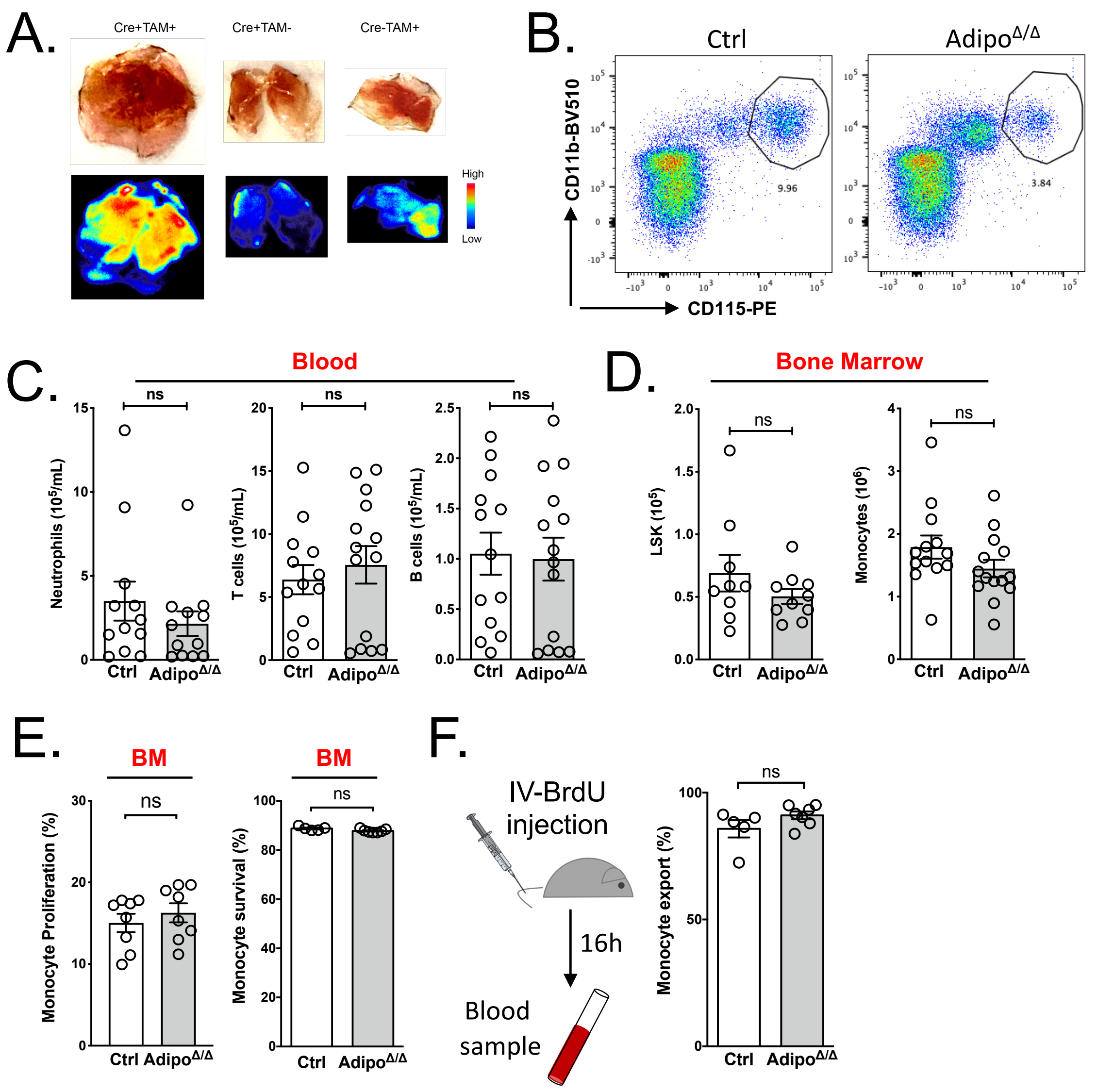

Figure S3. This figure is related to Figure 3.

(**A**) (Top panel) Representative photos showing the enlarged BAT in Adipo$^{\Delta/\Delta}$ mice compared to control groups 16 days post-tamoxifen administration. (Bottom panel) Representative autoradiography images showing the increased ^{64}Cu-DOTA-ECL1i signals in the BAT of Adipo$^{\Delta/\Delta}$ mice compared to control groups 16 days post-tamoxifen administration. (**B**) Representative dot plots of blood monocytes in control and Adipo$^{\Delta/\Delta}$ mice. (**C**) Quantification of blood neutrophil, T cell and B cell counts in control (n=12) and Adipo$^{\Delta/\Delta}$ (n=14) mice using flow cytometry. *p*=0,4428 (left), *p*=0,5826 (middle), *p*=0,6848 (right). (**D**) Quantification of bone marrow LSK (Lin$^-$ Sca1$^+$ cKit$^+$) and monocytes in control (n=9 and 13 respectively) and Adipo$^{\Delta/\Delta}$ (n=10 and 14 respectively) mice using flow cytometry. *p*=0,4002 (left) and *p*=0,0850 (right). (**E**) Bone marrow monocyte proliferation rate and survival in control (n=4 and n=5 respectively) and Adipo$^{\Delta/\Delta}$ (n=8 and n=7 respectively) mice by flow cytometry. *p*=0,3144 (left), *p*=0,0657 (right). (**F**) Schematic representation of Brdu injection protocol to visualize monocyte export from bone marrow to blood (left) and its quantification by flow cytometry in control (n=5) and Adipo$^{\Delta/\Delta}$ mice (n=7). *p*= 0,1490.

Panels C and D represent pooled data from 4 independent experiments. Panel E (left) represents pooled data from 2 independent experiments. Panels E (right) and F are representative of one experiment. All data are represented in means ± SEM. Two-tailed Mann Whitney tests were used to determine statistical significance. ns p>0,05 ; * p<0,05 ; ** p<0,01 ; *** p<0,001 ; **** p<0,0001. Source data are provided as a Source Data file.

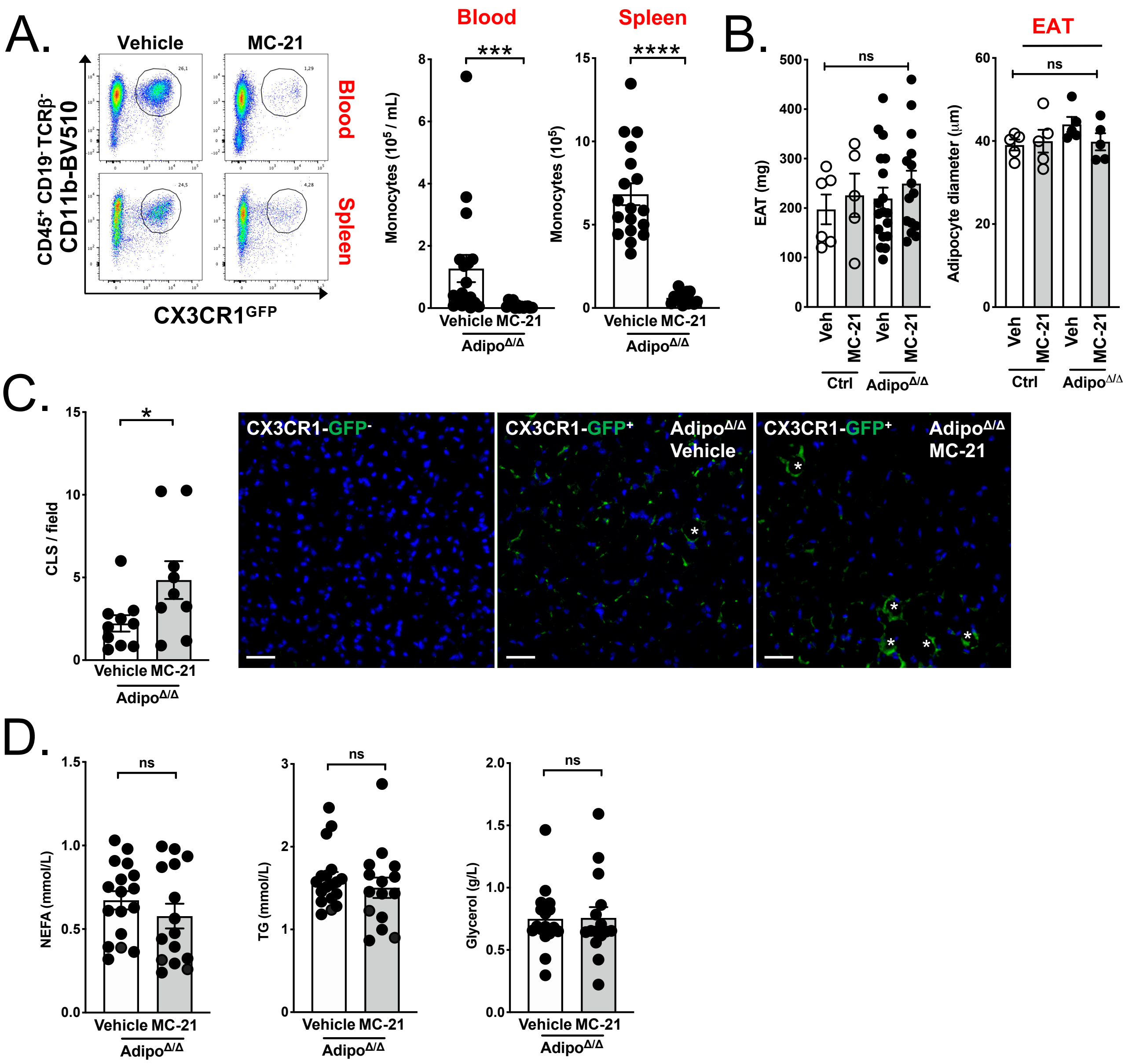

Figure S4. This figure is related to Figure 4.

(**A**) Representative dot plots (left) and quantification (right) of blood and spleen monocytes in MC-21 (n=13 and 15 respectively) or vehicle-treated (n=15 and 18 respectively) Adipo$^{\Delta/\Delta}$ mice. *p*= 0,0002 (left) and *p*<0,0001 (right). (**B**) (Left panel) EAT weight of MC-21 or vehicle-treated control (n=5 and 6 respectively) and Adipo$^{\Delta/\Delta}$ (n=15 and 18 respectively) mice and (right panel) EAT adipocyte diameter measurement. *p*>0,05 (all comparisons). (**C**) Quantification of crown-like structures (CLS) (left)) and immunofluorescence microscopy analysis in BAT of control (n=10) and MC-21-treated (n=9) Adipo$^{\Delta/\Delta}$ mice. *CX3CR1*$^{+/+}$ samples were used to determine background signal. White stars indicate crown-like structures. Scale bar = 100um. *p*=0,0364. (**D**) Glycerol, TG and NEFA levels in the serum of control (n=17) and MC-21-treated (n=15) Adipo$^{\Delta/\Delta}$ mice. *p*= 0,2948 (left), *p*= 0,5800 (middle) and *p*= 0,4474 (right).

Data were obtained from 3 pooled independent experiments. All data are represented in means ± SEM. Two-tailed Mann Whitney tests were used to determine statistical significance. ns p>0,05 ; * p<0,05 ; ** p<0,01 ; *** p<0,001 ; **** p<0,0001. Source data are provided as a Source Data file.

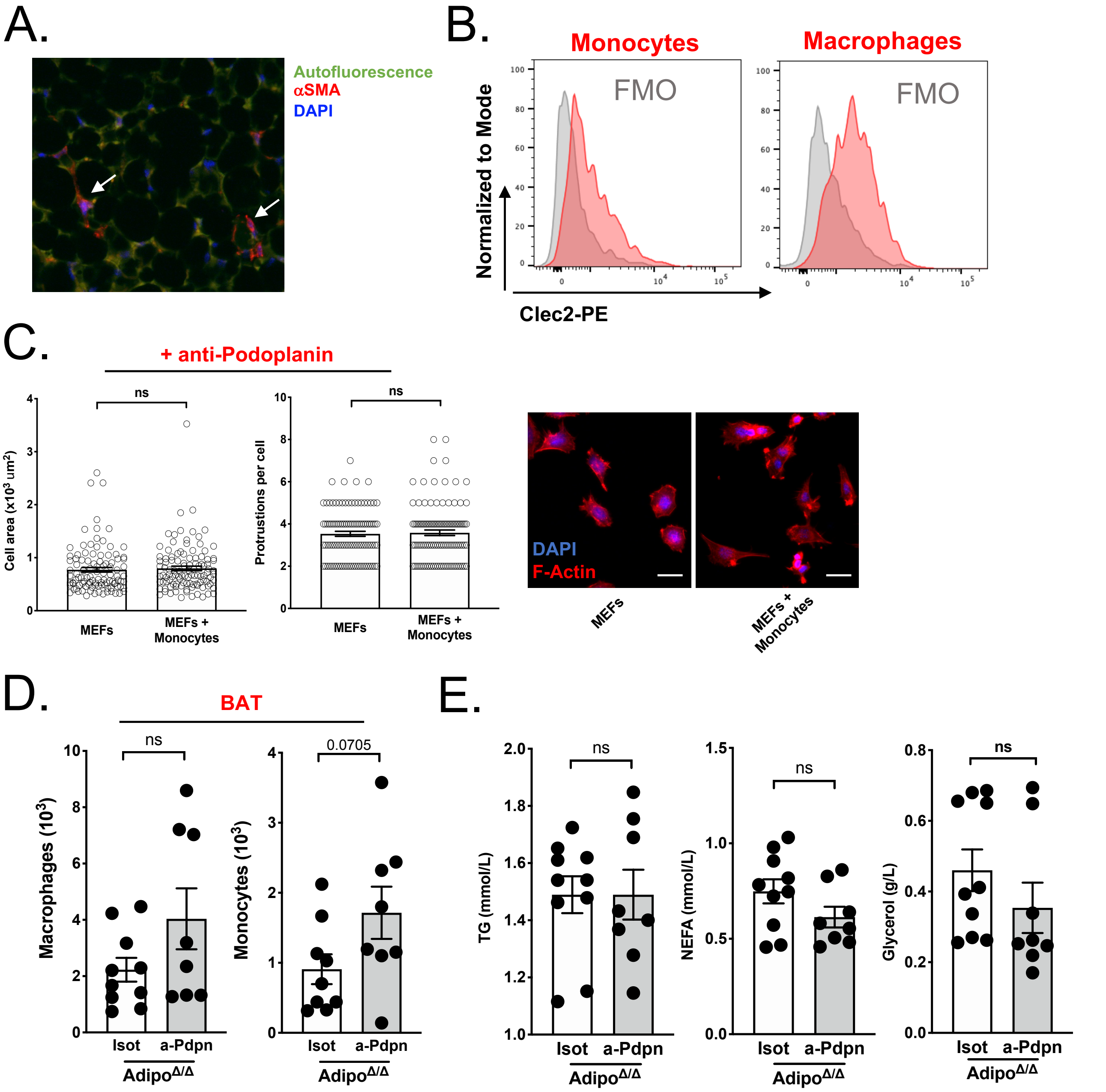

Figure S5. This figure is related to Figure 5.

(**A**) a-SMA staining (red) in the BAT of Adipo$^{\Delta/\Delta}$ mice. Autofluorescence signal (green) is used to show tissue architecture. Data representative of 2 independent experiments. (**B**) Histograms showing CLEC-2 and FMO stainings among BAT monocytes and macrophages from Adipo$^{\Delta/\Delta}$ mice. Data representative of 2 independent experiments. (**C**) (Left) Quantification of MEF morphological features after a 18hour co-culture experiment with monocytes in the presence of anti-Podoplanin blocking antibody. (Right) Representative images of MEF morphology at the end of the co-culture experiment. Scale bar = 20µm. *p*=0,3784 (left) and *p*=0,9203 (right). Data representative of 2 independent experiments. (**D**) Quantification of BAT macrophage and monocyte numbers in anti-Podoplanin (n=8) and isotype control-treated (n=10) Adipo$^{\Delta/\Delta}$ mice using flow cytometry. (**E**) Glycerol, TG and NEFA levels in the serum of isotype control (n=10) and anti-Podoplanin-treated (n=8) Adipo$^{\Delta/\Delta}$ mice. Data were obtained from 2 pooled independent experiments.

Panel C is representative of 2 independent experiments. Panels D and E represent pooled data from 2 pooled independent experiments. All data are represented in means ± SEM. Two-tailed Mann Whitney tests were used to determine statistical significance. ns p>0,05 ; * p<0,05 ; ** p<0,01 ; *** p<0,001 ; **** p<0,0001. Source data are provided as a Source Data file.

Supplementary Table 1

Primers used for genotyping and RT-qPCR.

**Mouse**	**Genotyping Primer sequences**	
Gene name	*Primer Forward (Left)*	*Primer Reverse (Right)*
GAPDH	*ACCACAGTCCATGCCATCACTGCCA*	*GGCCATCCACAGTCTTCTGC*
ß-Actin	*GAGACCTTCAACACCCC*	*GTGGTGGTGAAGCTGTAGGC*
CXCL12	*CCAAACTCTCCCCTTCAGAT*	*ATTTCGGGTCAATGCACACT*
CCL2	*CATCCACGTGTTGGCTCA*	*GATCATCTTGCTGGTGAATGAGT*
TNF-α	*CACAAGATGCTGGGACAGTGA*	*TCCTTGATGGTGGTGCATGA*

**Mouse**	**RT-qPCR Primer sequences**	
Gene name	*Primer Forward (Left)*	*Primer Reverse (Right)*
GAPDH	*ACCACAGTCCATGCCATCACTGCCA*	*GGCCATCCACAGTCTTCTGC*
ß-Actin	*GAGACCTTCAACACCCC*	*GTGGTGGTGAAGCTGTAGGC*
CXCL12	*CCAAACTCTCCCCTTCAGAT*	*ATTTCGGGTCAATGCACACT*
CCL2	*CATCCACGTGTTGGCTCA*	*GATCATCTTGCTGGTGAATGAGT*
TNF-α	*CACAAGATGCTGGGACAGTGA*	*TCCTTGATGGTGGTGCATGA*

Supplementary Table 2

List of reagents, materials, models and software used.

Reagent or resource	Source	Identifier
**Antibodies**		
CD115 PE (clone AFS98)	eBioscience	Cat# 12-1152-82
CD11b Brilliant Violet 510 (cloneM1/70)	Biolegend	Cat# 101263
Gr1 PerCP-Cy5.5 (clone RB6-8C5)	BD Biosciences	Cat# 552093
Ly6C PerCP-Cy5.5 (clone HK1.4)	Biolegend	Cat# 128011
F4/80 PE-Cy7 (clone BM8)	Biolegend	Cat# 123114
CD45 APC-Cy7 (clone 30-F11)	BD Biosciences	Cat# 557659
CD64 Brillant Violet 421 (clone X54-5/7.1)	Biolegend	Cat# 139309
CD19 FITC (clone 6D5)	Biolegend	Cat# 115506
CD301 FITC (clone ER-MP23)	Bio-Rad	Cat# MCA2392
MerTK PE (clone 2B10C42)	Biolegend	Cat# 151506
CD11c PE-Cy7 (clone HL3)	BD Biosciences	Cat# 558079
MHC II IA/IE APC (clone M5/114.15.2)	Biolegend	Cat# 107618
CD206 PerCp-Cy5.5 (clone C068C2)	Biolegend	Cat# 141715
CD206 AF647 (clone C068C2)	Biolegend	Cat# 141712
CD226 PerCp-Cy5.5 (clone10E5)	Biolegend	Cat# 128813
Clec2 PE (clone 17D9/CLEC-2)	Biolegend	Cat# 146103
TCRβ PB (clone H57-597)	Biolegend	Cat# 109226
CD3 APC (clone 17A2)	Biolegend	Cat# 100236
NK1.1 APC (clone PK136)	Biolegend	Cat# 108720
Ter119 APC (clone TER-119)	Biolegend	Cat# 116212
B220 APC (clone RA3-6B2)	BD Biosciences	Cat# 561226
CD19 APC (clone REA749)	Miltenyi Biotec	Cat# 130-111-884
CD150 PE-Cy7 (clone TC15-12F12.2)	Biolegend	Cat# 115914
Sca1 PB (clone D7)	Biolegend	Cat# 108120
c-Kit APC-Cy7 (clone ACK2)	eBioscience	Cat# 47-1172-82
CD48 AF488 (clone HM48-1)	Biolegend	Cat# 103414

CXCR4 APC (clone 2B11)	eBioscience	Cat# 51-9991-80
CD11b APC (clone M1/70)	Biolegend	Cat# 101218
MC-21	Dr Mack M.	
Podoplanin (clone 8.1.1)	BioXCell	Cat# BE0236
*InVivo*MAb polyclonal Syrian hamster IgG	BioXCell	Cat# BE0087
Ly6G Biotin (clone REA526)	Miltenyi biotec	Cat# 130-116-512
CD3 Biotin (clone REA641)	Miltenyi biotec	Cat# 130-123-861
B220 Biotin (clone REA755)	Miltenyi biotec	Cat# 130-110-844
NK1.1 Biotin (clone REA1162)	Miltenyi biotec	Cat# 130-120-513
Ter-119 Biotin (clone Ter-119)	Miltenyi biotec	Cat# 130-120-828
SiglecF Biotin (clone REA798)	Miltenyi biotec	Cat# 130-112-329
α-SMA	Abcam	Cat# ab5694
Goat anti Rabbit Cy3	Jackson ImmunoResearch	Cat# 111-165-003

Chemicals

DAPI	Sigma	Cat# D9542
Phalloidin Texas Red	Invitrogen	Cat# T7471
PFA 4%	VWR International	Cat# 9713.1000
Bovine serum Albumin (BSA)	Sigma	Cat# A7030
Tamoxifen	Sigma	Cat# T5648
RPMI medium	Life Technologies	Cat# 21875091
DMEM medium	Life Technologies	Cat# 11960044
Collagenase A	Sigma	Cat# 11088793001
IHC Antigen retrieval solution	eBiosciences	Cat# 00-4955-58
ImmunoHistoMount	Sigma	Cat# I1161
Thiazolyl blue tetrazolium bromide	Sigma	Cat# M2128
Fetal bovine serum	Fisher Scientific	Cat# 12350273
Lysing buffer	BD Biosciences	Cat# 555899
L-Glutamine	Life Technologies	Cat# 25030024
Penicillin Streptomycin	Life Technologies	Cat# 15070063
Sodium Pyruvate	Life Technologies	Cat# 11360039
Free glycerol reagent	Sigma	Cat# F6428
Power SYBR^{TM} green PCR Master Mix	Applied Biosystems	Cat# 4367659
RIPA buffer	Cell signaling	Cat# 9806
Anti-Biotin MicroBeads	Miltenyi Biotec	Cat# 130-090-858

Critical commercial Assays

High-Capacity cDNA reverse transcription kit	Applied Biosystems	Cat# 4368814
CCL2 DuoSet ELISA	R&D Systems	Cat# DY479-05
TNF-alpha DuoSet ELISA	R&D Systems	Cat# DY410-05
RNeasy Plus Mini Kit (250)	QIAGEN	Cat# 74136
Mouse CXCL12/SDF-1 DuoSet ELISA	R&D Systems	Cat# DY460
(Stromal Cell Derived Factor 1), Human, Elisa Kit	EUROMEDEX	Cat# EH3755
NEFA-HR2 R1 + R2 FUJFILM	WAKO	Cat# W1W270-77000
Glucose dosage Kit	BioSentec	Cat# 075
Triglyceride dosage Kit	DiaSys	Cat# 157109910021
BrdU APC Staining Kit	ThermoFisher	Cat# 8817-6600-42
0.4 μm Cell culture Inserts 24 well format	Falcon	Cat# 353095

Experimental models: Organisms/Strains

Mouse: C57BL/6-Tg(Adipoq-cre/ERT2)1Soff/J	The Jackson laboratory	JaxStock# 025124
Mouse: B6.FVB-Tg(Ucp1-cre)1Evdr/J	The Jackson laboratory	JaxStock# 024670
Mouse: B6N.129S-Pnpla2^{tm1Eek}/J	The Jackson laboratory	JaxStock# 024278
Mouse: B6.129P-Cx3cr1^{tm1Litt}/J	The Jackson laboratory	JaxStock# 008451
Mouse: B6.Cg-Gt(ROSA)26Sor$^{tm9(CAG-tdTomato)Hze}$/J	The Jackson laboratory	JaxStock# 007909
Mouse: C57BL/6NTac-Ccr2^{tm2982}(T2A-Cre7ESR1-T2A-mKate2]	Dr. Burkhard Becher	N/A
Mouse: CCR2^{GFP}	Dr. Marco Colonna	N/A
Cell line : Mouse Embryonic Fibroblasts (MEFs)	ATCC	CRL-2907

Accessories

StepOne	Applied Biosystem	N/A
Thermo Cycler SimpliAmp	Applied Biosystem	N/A
Nanodrop	OZYME	
MACS Multistand	Miltenyi Biotec	Cat# 130-042-303
MACS LS Columns	Miltenyi Biotec	Cat# 130-042-401

Softwares

Prism6	GraphPad	N/A
FlowJo	Tree Star	N/A
Fuji	Fiji	N/A
GENEsys	Syngene	N/A
StepOne Software v.2.2.2	Applied Biosystem	N/A
Fiji	https://imagej.net/software/fiji/	N/A
