# Supplementary material for: Brown adipose tissue monocytes support tissue expansion
Source: Nat Commun. 2021 Sep 6;12:5255. doi: 10.1038/s41467-021-25616-1 (PMC8421389; doi:10.1038/s41467-021-25616-1)
Supplement: Supplementary file 3 — Reporting Summary [file 41467_2021_25616_MOESM3_ESM.pdf]

Corresponding author(s): Stoyan Ivanov

Last updated by author(s): 16/06/2021

Reporting Summary

Nature Research wishes to improve the reproducibility of the work that we publish. This form provides structure for consistency and transparency in reporting. For further information on Nature Research policies, see our Editorial Policies and the Editorial Policy Checklist.

Statistics

For all statistical analyses, confirm that the following items are present in the figure legend, table legend, main text, or Methods section.

n/a	Confirmed	
☐	☒	The exact sample size (*n*) for each experimental group/condition, given as a discrete number and unit of measurement
☐	☒	A statement on whether measurements were taken from distinct samples or whether the same sample was measured repeatedly
☐	☒	The statistical test(s) used AND whether they are one- or two-sided<br>*Only common tests should be described solely by name; describe more complex techniques in the Methods section.*
☒	☐	A description of all covariates tested
☐	☒	A description of any assumptions or corrections, such as tests of normality and adjustment for multiple comparisons
☐	☒	A full description of the statistical parameters including central tendency (e.g. means) or other basic estimates (e.g. regression coefficient) AND variation (e.g. standard deviation) or associated estimates of uncertainty (e.g. confidence intervals)
☐	☒	For null hypothesis testing, the test statistic (e.g. *F*, *t*, *r*) with confidence intervals, effect sizes, degrees of freedom and *P* value noted<br>*Give P values as exact values whenever suitable.*
☒	☐	For Bayesian analysis, information on the choice of priors and Markov chain Monte Carlo settings
☒	☐	For hierarchical and complex designs, identification of the appropriate level for tests and full reporting of outcomes
☒	☐	Estimates of effect sizes (e.g. Cohen's *d*, Pearson's *r*), indicating how they were calculated

Our web collection on statistics for biologists contains articles on many of the points above.

Software and code

Policy information about availability of computer code

Data collection	Flow Cytometry Data were collected on a BD Facs CANTO II using BD Diva software.<br><br>scRNA libraries were sequenced on an Illumina NextSeq 500/550 High Output flowcell: the forward read had a length of 28 bases that included the cell barcode and the UMI; the reverse read had a length of 55 bases that contained the cDNA insert. Alignment, barcode assignment and UMI counting with Cell Ranger v4.0.0 was used to perform sample demultiplexing, barcode processing and single-cell 3' counting. Cell Ranger's mkfastq function was used to demultiplex raw base call files from the HiSeq4000 sequencer into sample specific FASTQ files.<br><br>Traction Force Microscopy : Images of gel surface– conjugated fluorescent beads were acquired for each cell before and after cell removal using a Axiovert 200M motorized microscope stand (Zeiss) and a ×32 magnification objective.<br><br>For PET/CT, 45 to 60 minutes dynamic scan was performed after injection of 64Cu-DOTA984 ECL1i (3.7 MBq in 100 µL saline) via tail vein with Inveon PET/CT system (Siemens, Malvern, 985 PA).<br><br>qPCRs were performed on the StepOne device (Applied Biosystem).
Data analysis	Microsoft Excel V16.<br>Flowjo analysis software V10, Treestar.

Graphpad Prism V8.
Fiji analysis software V2.

The PET images were reconstructed with the maximum a posteriori algorithm and analyzed by Inveon Research Workplace. Organs of interest were collected, weighed, and counted in a Beckman 8000 gamma counter (Beckman, Fullerton, CA). After the collection of BATs, the radioactivities in the tissues were detected by autoradiograph using a Storm Phosphorimager (GE, Marlborough, MA).

Single Cell RNA sequencing :
DropletUtils package (version 1.4.3),
Seurat package (version 3.1.0)
Dyno package (version 0.1.2)
Slingshot singularity container (version 1.0.3).

Tractions exerted by MEF were estimated by measuring bead displacement fields, computing corresponding traction fields using Fourier transform traction microscopy, and calculating root mean-square traction using the PIV (particle Image velocity) and TFM (Traction force microscopy) pakage on ImageJ.

For manuscripts utilizing custom algorithms or software that are central to the research but not yet described in published literature, software must be made available to editors and reviewers. We strongly encourage code deposition in a community repository (e.g. GitHub). See the Nature Research guidelines for submitting code & software for further information.

Data

Policy information about availability of data

All manuscripts must include a data availability statement. This statement should provide the following information, where applicable:
- Accession codes, unique identifiers, or web links for publicly available datasets
- A list of figures that have associated raw data
- A description of any restrictions on data availability

Gene expression data (scRNA-seq) have been uploaded to the Gene Expression Omnibus (GEO) repository for public availability under the accession code GSE177635 and can be explored here. All other data supporting the findings of this study are available from the corresponding author upon reasonable request. Source data are provided with this paper.

Field-specific reporting

Please select the one below that is the best fit for your research. If you are not sure, read the appropriate sections before making your selection.

- [x] Life sciences
- [ ] Behavioural & social sciences
- [ ] Ecological, evolutionary & environmental sciences

For a reference copy of the document with all sections, see nature.com/documents/nr-reporting-summary-flat.pdf

Life sciences study design

All studies must disclose on these points even when the disclosure is negative.

Sample size	No sample size calculations were performed. To comply with the principles of the 3Rs (Replacement, Reduction and Refinement), we have used minimal sample size sufficient to detect biological differences. Numbers of samples are indicated in the figure legends for each panel.
Data exclusions	Statistical outliers were indentified using Grubb's test on the GraphPad Prism software.
Replication	The number of biologically-independent samples is indicated in figure legends corresponding to each panel.
Randomization	Mice were age-matched for in vivo experiment. Mice were randomly attributed to each experimental group when possible, depending on their genotype. Furthermore, if possible, animals were co-housed. In ex vivo experiments samples were also matched for age and sex. For in vitro experiments all allocations were random.
Blinding	For tissue histology and cellular microscopy analyses, images were attributed a number by the experimentator (who was aware of the genotype and treatment used) and analyzed in a blind fashion by another member of the group who was unaware of the experimental parameters. Correspondance was revealed after image analysis. For other experiments (flow cytometry...), the experimentator was not blinded to the genotype and treatment as these informations were needed and known in order to start treatments and prepare the samples.

Reporting for specific materials, systems and methods

We require information from authors about some types of materials, experimental systems and methods used in many studies. Here, indicate whether each material, system or method listed is relevant to your study. If you are not sure if a list item applies to your research, read the appropriate section before selecting a response.

Materials & experimental systems

n/a	Involved in the study
[ ]	[x] Antibodies
[ ]	[x] Eukaryotic cell lines
[x]	[ ] Palaeontology and archaeology
[ ]	[x] Animals and other organisms
[x]	[ ] Human research participants
[x]	[ ] Clinical data
[x]	[ ] Dual use research of concern

Methods

n/a	Involved in the study
[x]	[ ] ChIP-seq
[ ]	[x] Flow cytometry
[x]	[ ] MRI-based neuroimaging

Antibodies

Antibodies used

CD115 PE (clone AFS98) eBioscience Cat# 12-1152-82
CD11b Brilliant Violet 510 (cloneM1/70) Biolegend Cat# 101263
Gr1 PerCp-Cy5.5 (clone RB6-8C5) BD Biosciences Cat# 552093
F4/80 PE-Cy7 (clone BM8) Biolegend Cat# 123114
CD45 APC-Cy7 (clone 30-F11) BD Biosciences Cat# 557659
CD64 Brillant Violet 421 = FcgRI) (clone X54-5/7.1) Biolegend Cat# 139309
CD19 FITC (clone 6D5) Biolegend Cat# 115506
CD301 FITC (clone ER-MP23) Bio-Rad Cat# MCA2392
MerTK PE (clone 2B10C42) Biolegend Cat# 151506
CD11c PE-Cy7 (clone HL3) BD Biosciences Cat# 558079
Ly6C-PerCP-Cy5.5 (clone HK1.4) Biolegend Cat#128011
MHC II IA/IE APC (clone M5/114.15.2) Biolegend Cat# 107618
CD206 PerCp-Cy5.5 (clone C068C2) Biolegend Cat# 141715
CD206 AF647 (clone C068C2) Biolegend Cat# 141712
CD226 PerCp-Cy5.5 (clone10E5) Biolegend Cat# 128813
Clec2 PE (clone 17D9/CLEC-2) Biolegend Cat# 146103
TCRb PB (clone H57-597) Biolegend Cat# 109226
CD3 APC (clone 17A2) Biolegend Cat# 100236
NK1.1 APC (clone PK136) Biolegend Cat# 108720
Ter119 APC (clone TER-119) Biolegend Cat# 116212
B220 APC (clone RA3-6B2) BD Biosciences Cat# 561226
CD19 APC (clone REA749) Miltenyi Biotec Cat# 130-111-884
CD150 PE-Cy7 (clone TC15-12F12.2) Biolegend Cat# 115914
Sca1 PB (clone D7) Biolegend Cat# 108120
c-Kit APC-Cy7 (clone ACK2) eBioscience Cat# 47-1172-82
CD48 AF488 (clone HM48-1) Biolegend Cat# 103414
CXCR4 APC (clone 2B11) eBioscience Cat# 51-9991-8033
CD11b APC (clone M1/70) Biolegend Cat# 101218
MC-21 (not commercially available) was provided by Dr Mack M.
Podoplanin (clone 8.1.1) BioXCell Cat# BE0236
InVivoMAb polyclonal Syrian hamster IgG BioXCell Cat# BE0087
Ly6G Biotin (clone REA526) Miltenyi biotec Cat# 130-116-512
CD3 Biotin (clone REA641) Miltenyi biotec Cat# 130-123-861
B220 Biotin (clone REA755) Miltenyi biotec Cat# 130-110-844
NK1.1 Biotin (clone REA1162) Miltenyi biotec Cat# 130-120-513
Ter-119 Biotin (clone Ter-119) Miltenyi biotec Cat# 130-120-828
SiglecF Biotin (clone REA798) Miltenyi biotec Cat# 130-112-329
a-SMA Abcam Cat# ab5694
Goat anti Rabbit Cy3 Jackson ImmunoResearch Cat# 111-165-003

Validation

Anti-CCR2 antibody (MC-21) was provided by Dr. Matthias Mack. The validation of this antibody was previously performed (Mack et al., J Immunol 2001, cited in the manuscript).
All other antibodies were obtained from commercial sources and validation data as well as detailed information can be found on the manufacturer's website (Biolegend, eBioscience, BD Biosciences, Miltenyi, BioXcell, Abcam, Jackson ImmunoResearch).

Eukaryotic cell lines

Policy information about cell lines

Cell line source(s)

Mouse Embryonic Fibroblasts (WT SV40 MEF) were obtained from ATCC (CRL-2907) and used within 30 passages after thawing.

Authentication

Further authentication was not performed.

Mycoplasma contamination	None.
Commonly misidentified lines (See ICLAC register)	No commonly misidentified lines were used in the study.

Animals and other organisms

Policy information about studies involving animals; ARRIVE guidelines recommended for reporting animal research

Laboratory animals	Both male and female mice were used between 6 and 20 week of age. Pnpla2fl/fl [B6N.129S-Pnpla2tm1Eek/J], CX3CR1gfp [B6.129P-Cx3cr1tm1Litt/J], and TdTomatofl/fl [B6.Cg-Gt(ROSA)26Sortm9(CAG-tdTomato)Hze/J] mice were crossed together and then crossed to AdipoQcreERT2 [C57BL/6-Tg(Adipoq-cre/ERT2)1Soff/J] or Ucp1Cre [B6.FVB-Tg(Ucp1-cre)1Evdr/J] mice (all mice were purchased from The Jackson Laboratory; Ucp1cre mice were kindly provided by Dr. Jean-François Tanti). CCR2creERT2 [C57BL/6NTac-Ccr2tm2982(T2ACre7ESR1-T2A-mKate2)] mice were kindly provided by Dr. Burkhard Becher and crossed to TdTomatofl/fl and CCR2GFP [B6(C)-Ccr2tm1.1Cln/J] provided by Dr. Marco Colonna. Mice were housed under 12h light/dark cycles and at ambient temperature between 20 and 23°C with 55-65% humidity.
Wild animals	No wild animals were used in this study.
Field-collected samples	No field-collected samples were used in this study.
Ethics oversight	For each experiment, co-housed littermate controls were used. Animal protocols were approved by the Institutional Animal Care and Use Committee of the French Ministry of Higher Education and Research and the Mediterranean Center of Molecular Medicine (INSERM U1065) and were undertaken in accordance with the European Guidelines for Care and Use of Experimental Animals.

Note that full information on the approval of the study protocol must also be provided in the manuscript.

Flow Cytometry

Plots

Confirm that:

- [x] The axis labels state the marker and fluorochrome used (e.g. CD4-FITC).
- [x] The axis scales are clearly visible. Include numbers along axes only for bottom left plot of group (a 'group' is an analysis of identical markers).
- [x] All plots are contour plots with outliers or pseudocolor plots.
- [x] A numerical value for number of cells or percentage (with statistics) is provided.

Methodology

Sample preparation	Blood analysis<br>Just before the animal sacrifice, few drops of blood were collected by submandibular bleeding. Lysing buffer was used for red blood cells lysis. Cells were then centrifuged (400g, 5min at 4°C) and stained for 25 min protected from light in FACS buffer (RPMI medium, 0,3mM EDTA, and 0,06% BSA). Cells were then washed, centrifuged and data were acquired on BD FACS Canto flow cytometer. Analysis was performed using FlowJo software (Tree Star).<br>Tissue Analysis<br>Adipose tissues were harvested, shredded with scissors and then incubated for 30 minutes with PBS containing 1.5 mg/ml collagenase A at 37°C. Digested adipose tissue were homogenized using a 1mL syringe with a 20G needle and passed through a 100µm sieve. Spleens were crushed with a piston in PBS through a 100µm sieve. The resulting suspension was lysed and processed as described in the previous paragraph. Femurs and tibias were harvested and flushed with FACS buffer. Cells were then centrifuged (400g, 5min at 4°C), lysed and stained as previously described.
Instrument	BD Facs CANTO II
Software	FACS Diva software.<br>Analysis : Flowjo
Cell population abundance	Cell sorting was performed using a BD Facs Aria III. Single (gated using FSC-A/FSC-H), Live (Dapi negative) CD45+ cells were cell sorted and submitted to Single Cell RNA sequencing. The gating strategy used is described in supplementary figure 1A. The quality of the preparation was evaluated by Ptprc expression profile in our scRNA-seq data.
Gating strategy	Gating strategies used in the present study are described in corresponding supplemental panels (S1A, S2C, S2D, S3B, S4A).<br>For CCR2 pulse-chase experiments, myeloid cells were identified as CD45+ CD11b+ CD64+ cells. Macrophages were further identified as CD45+ CD11b+ CD64+ Ly6C- cells. For the rest of the study, macrophages were identified as CD45+ CD64+ MerTK+ cells and monocytes as CD45+ MerTK- CD64+ CD11b+ cells. In CX3CR1-GFP mice, CX3CR1 was further used to identify monocytes. For blood samples, monocytes were identified as CD45+ CD11b+ CD115+ cells, neutrophils as CD45+ CD11b+ CD115- Gr1+ cells, B cells as CD45+ CD19+ and T cells as CD45+ TCRb+.

- [x] Tick this box to confirm that a figure exemplifying the gating strategy is provided in the Supplementary Information.
